# The aggregate value of cancer screenings in the United States: full potential value and value considering adherence

**DOI:** 10.1186/s12913-023-09738-4

**Published:** 2023-08-07

**Authors:** Tomas J. Philipson, Troy Durie, Ze Cong, A. Mark Fendrick

**Affiliations:** 1 University of Chicago, Chicago, IL USA; 2 GRAIL, LLC, a subsidiary of Illumina, Inc., currently held separate from Illumina Inc., under the terms of the Interim Measures Order of the European Commission dated 29 October 2021, Menlo Park, CA USA; 3 University of Michigan, Ann Arbor, MI USA

**Keywords:** Cancer screening, Life-years gained, Value of cancer screening, Multi-cancer early detection

## Abstract

**Background:**

Although cancer mortality has been decreasing since 1991, many cancers are still not detected until later stages with poorer outcomes. Screening for early-stage cancer can save lives because treatments are generally more effective at earlier than later stages of disease. Evidence of the aggregate benefits of guideline-recommended single-site cancer screenings has been limited. This article assesses the benefits in terms of life-years gained and associated value from major cancer screening technologies in the United States.

**Methods:**

A mathematical model was built to estimate the aggregate benefits of screenings for breast, colorectal, cervical, and lung cancer over time since the start of US Preventive Services Task Force (USPSTF) recommendations. For each type, the full potential benefits under perfect adherence and the benefits considering reported adherence rates were estimated. The effectiveness of each screening technology was abstracted from published literature on the life-years gained per screened individual. The number of individuals eligible for screening per year was estimated using US Census data matched to the USPSTF recommendations, which changed over time. Adherence rates to screening protocols were based on the National Health Interview Survey results with extrapolation.

**Results:**

Since initial USPSTF recommendations, up to 417 million people were eligible for cancer screening. Assuming perfect adherence to screening recommendations, the life-years gained from screenings are estimated to be 15.5–21.3 million (2.2–4.9, 1.4–3.6, 11.4–12.3, and 0.5 million for breast, colorectal, cervical, and lung cancer, respectively). At reported adherence rates, combined screening has saved 12.2–16.2 million life-years since the introduction of USPSTF recommendations, ~ 75% of potential with perfect adherence. These benefits translate into a value of $8.2-$11.3 trillion at full potential and $6.5-$8.6 trillion considering current adherence. Therefore, single-site screening could have saved an additional 3.2–5.1 million life-years, equating to $1.7-$2.7 trillion, with perfect adherence.

**Conclusions:**

Although gaps persist between the full potential benefit and benefits considering adherence, existing cancer screening technologies have offered significant value to the US population. Technologies and policy interventions that can improve adherence and/or expand the number of cancer types tested will provide significantly more value and save significantly more patient lives.

**Supplementary Information:**

The online version contains supplementary material available at 10.1186/s12913-023-09738-4.

## Introduction

Approximately 1.9 million new cancer cases and 609,360 cancer deaths are projected in the United States during 2022 [1]. Cancer mortality has declined significantly in the United States in recent decades: between 1991 and 2019, the cancer death rate decreased 32%, translating to 3.4 million fewer deaths during this period [1]. This has been accomplished through public health measures, such as reduction in smoking, more efficacious cancer treatments, and better and expanded screenings that allow cancer diagnosis at earlier stages [1, 2].

**Fig. 1:**
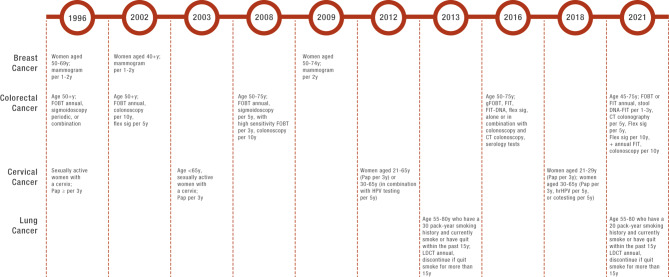
Screening Protocols Recommended by the USPSTF Over Time for Four Cancer Types CT, computed tomography; FIT, fecal immunochemical test; Flex sig, flexible sigmoidoscopy; FOBT, fecal occult blood test; gFOBT, guaiac fecal occult blood test; HPV, human papilloma virus; hrHPV, high-risk human papillomavirus; LDCT, low-dose computed tomography; Pap, Papanicolaou smear or Pap test; USPSTF, US Preventive Services Task Force

Currently, the US Preventive Services Task Force (USPSTF) recommends single-site cancer screening for breast, colorectal, cervical, and lung cancer for at-risk individuals [3–6]. General screening recommendations include mammograms for breast cancer detection; high-sensitivity stool-based tests, colonoscopy, computed tomography (CT) colonography, or flexible sigmoidoscopy for colon cancer; high-risk human papillomavirus and/or cytology for cervical cancer; and low-dose CT for lung cancer detection [3–6]. The eligibility criteria of a given screening population, types of screening technologies recommended, and screening intervals have evolved over time. Each test has its own unique USPSTF-recommended population based on age, sex, and, in the case of lung cancer, history of smoking (Fig. 1). For example, mammography was first recommended by USPSTF as screening for breast cancer in 1996 for women aged 50 to 69 years with a screening frequency of once per 1 to 2 years [7]. In 2002, the recommended age group was changed to women aged ≥ 40 years [8], and in 2009, the recommendation was further adjusted to be among women aged 50 to 74 years with a screening frequency of once per 2 years [9]. In addition, recommendations for prostate cancer screening testing such as the prostate specific antigen (PSA) test have changed over time. PSA was not recommended by USPSTF for men aged ≥ 70 years old; men ages 55 to 69 years were recommended to discuss the possible benefits and harms of PSA screening with their health care provider to make an individualized decision about whether to get screened [10]. Conversely, other medical societies such as the American Cancer Society (ACS) have recommended men aged ≥ 50 years with average risk and men aged ≥ 40 years with higher risk for prostate cancer to be screened by PSA annually since 1992 [11].

Randomized controlled trials, observational studies, and case-cohort studies have demonstrated that screening tests for breast, colorectal, cervical, and lung cancer can increase life span through early detection and treatment [3, 5, 12–14]. Current reports suggest that early diagnosis not only reduces cancer-related mortality but may result in a cancer treatment cost reduction of $26 billion per year in the United States and reduce financial impact on patients and their families [15, 16]. However, not all types of cancer have screening tests, and barriers remain with respect to current testing modalities [17]. Cancers without a USPSTF-recommended screening paradigm account for almost 70% of cancer deaths in the United States [18, 19].

Based on survey data, screenings were increasingly adopted after being recommended by USPSTF. However, not all patients undergo recommended screening. The Healthy People 2020 targets for cancer screening, provided by the US Department of Health and Human Services Office of Disease Prevention and Health Promotion, are 70.5% for colorectal, 81.1% for breast, and 93% for cervical cancers among those eligible for screening [20, 21]. However, screening rates have remained below these targets: although approximately 83% of the eligible population underwent recommended screening for cervical cancers, only 72% were up-to-date with screening for breast cancer and 67% underwent recommended screening for colorectal cancer based on self-reported survey data [22]. In real-world practice, individuals’ demographic characteristics, including socioeconomic status, age, personal attitudes, beliefs, awareness, access to healthcare facilities, and social support; as well as physicians’ recommendations on cancer screenings, inconsistency of USPSTF-recommended eligibility criteria over time, and differences among guidelines can all influence adherence to screening recommendations [23].

To date, studies assessing survival benefits of cancer screenings have been either based on large-scale randomized controlled trials [24] that reported mortality rate reduction in terms of incidence rate reduction (IRR), or based on cohort or simulation modeling as part of a cost-effectiveness assessment reporting life-years gained per screened individual [22–25]. Both approaches provide estimations of the survival benefit of a specific cancer screening technology at the individual level. Limited literature has examined the population-level, longitudinal, aggregate survival benefits of cancer screenings following USPSTF recommendations considering the *total number of eligible individuals* and the *total number of individuals receiving* the screenings. This information will be critical for policy decision makers to comprehensively assess the overall value of cancer screenings.

This research sought to quantify the aggregate gains to US life expectancy, to date, since major screening technologies for 4 cancer types were recommended by the USPSTF. The gains from using PSA to screen for prostate cancer as recommended by ACS were also quantified.

## Methods

**Fig. 2:**
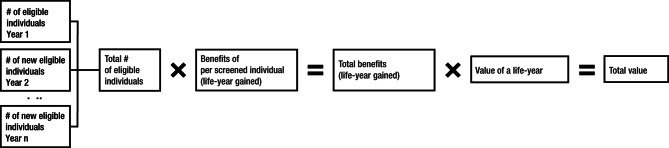
Methodology to Estimate the Full Potential Value of Cancer Screening

The full potential clinical and economic value of recommended screenings for breast, colorectal, cervical, and lung cancers from 1996 (2013 for lung cancer) to 2020 were estimated with a mathematical model (Fig. 2), assuming all eligible individuals were perfectly adherent to their recommended screening protocols. This was accomplished by calculating the total number of eligible individuals in the first year of each USPSTF recommendation using US Census data. The numbers of individuals who newly became eligible in subsequent years were also estimated using US Census data. The total number of individuals eligible for screening over time was calculated by taking the summation of the numbers of eligible individuals across all birth cohorts. The effectiveness of breast, colorectal, cervical, lung, and prostate screenings in terms of life-years gained per screened individual was obtained from a targeted literature review of the cost-effectiveness of the screening technologies [25–29] (Supplemental Table 1); underlying assumptions about natural history, cancer incidence, stage-specific survival, test characteristics, costs, etc. are reported in the collected articles. The total number of life-years gained was calculated by multiplying the total number of eligible individuals for each cancer screening by the number of life-years gained per screened individual from that screening. The full economic potential value of cancer screenings was calculated by applying the value of a life-year (VLY; $531,501 based on systematic review) to the estimated full potential life-years gained [30] (Fig. 2). A sensitivity analysis was performed using a more conservative VLY of $150,000.

**Fig. 3:**
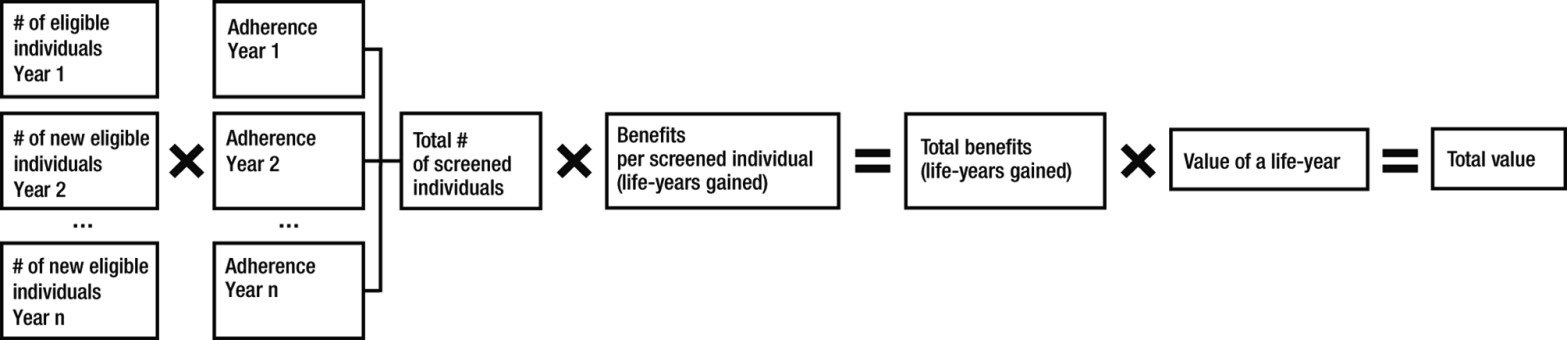
Methodology to Estimate the Value of Cancer Screening Considering Adherence

To estimate the value of cancer screening considering adherence (Fig. 3), the total number of eligible individuals was weighted by the adherence rates for each cancer screening for each year [31–33]. We used publicly available data sources from the National Health Interview Survey (NHIS) for the cancer screening adherence rates [34] and imputed the adherence rates in each year using a linear extrapolation.

To provide a range estimation of the total clinical benefits and the economic value that covers the various test recommendations and screening populations, a minimum and a maximum scenario were considered in calculating life-years gained and the associated value. Because both the screening eligibility and the technologies recommended by USPSTF changed over time, a minimum scenario was assessed using the most restrictive screening population (smallest possible eligible cohort) that the USPSTF ever recommended and the lowest effectiveness estimated in the literature; a maximum scenario was assessed using the least restrictive screening population (largest possible eligible cohort) and the largest effectiveness estimated (Table 1). The benefits from using PSA to screen for prostate cancer according to ACS recommendation were also estimated following the same methodology as the other 4 cancer types, starting from 1992.

**Table 1:** Eligible Population and Effectiveness Assumptions for Minimum and Maximum Scenarios

Cancer Type	Minimum Scenario			Maximum Scenario		
	1st Year USPSTF Recommendation	Eligible Population	Life-Years Gained per Screened Individual	1st Year USPSTF Recommendation	Eligible Population	Life-Years Gained per Screened Individual
Breast	1996	Women; age 50–69 y	0.029 [22]	1996	Women, age 40 + y	0.044 [22]
Colorectal	1996	Age 50–75 y	Flex sig + FIT^a^: 0.009 [23]	2002	Age 45–75 y	Colonoscopy: 0.022 [23]
Cervical	1996	Women; age 21–65 y	PAP^b^: 0.089 [24]	1996	Women; age 21–65 y	21–30 PAP; 30–65 cotesting^c^: 0.096 [24]
Lung	2014	Current smoker or former smoker; age 50–80 y	0.0316 [25]	Limited benefit evidence, same as minimum scenario		

^a^Flex sig: flexible sigmoidoscopy, an endoscopic procedure that allows physician to examine the rectum and lower colon

^b^PAP: a Pap smear or Pap test is a screening test for cervical cancer

^c^Cotesting: combination of PAP and human papilloma virus testing

FIT, fecal immunochemical test; USPSTF, US Preventive Services Task Force

### Data sources

No new human data were collected in this study, and all data analyzed in the manuscript are publicly available (direct references to source data are included in Supplemental Table 2). The size of each screening cohort was determined by the number of individuals in the population who met the sex (for breast and cervical) and age criteria for that cohort. The US Census Bureau’s Vintage annual national population estimates by demographic characteristics and Census’s National Intercensal Datasets were used to determine the size of the eligible population for each screening cohort over time. These data estimate the population in the United States each year between the decennial census, which stratifies data by sex and by single year of age. For example, the dataset reports how many 65-year-olds are in the country at any given year.

Lung cancer screenings were recommended for all adults aged 55 to 80 years since 2013, with a ≥ 30 pack-year cigarette smoking history and currently smoking or having quit < 15 years ago. This was estimated to be 12.7% of adults aged 55 to 80 years in 2017 based on the Behavioral Risk Factor Surveillance System (BRFSS) survey [32]. This percentage was applied to the number of adults estimated to be aged 55 to 80 years in the census data to estimate the number of eligible individuals for lung cancer screening.

The colorectal cancer minimum scenario started in 1996, because the original test, annual fecal occult blood test and/or period sigmoidoscopy, started in that year with relatively lower effectiveness. Conversely, the colorectal cancer maximum scenario started in 2002, when colonoscopy became the recommended test for this cancer type with the largest effectiveness among all the screening technologies recommended.

Adherence rates were obtained from the NHIS for breast cancer, colorectal cancer, and cervical cancer screening for the years 2008, 2010, 2013, 2015, and 2018. Lung cancer screening compliance rates were obtained from the BRFSS survey [32, 33]. Adherence rates for prostate cancer screening were based on NHIS datasets for the years 1999, 2000, 2003, 2005, 2008, 2010, 2013, 2015, and 2018 [35].

Because adherence rates were not reported for each year in the dataset, a linear trend imputation was calculated based on the observed adherence rates for breast cancer, colorectal cancer, cervical cancer, and prostate cancer. Lung cancer adherence rates were only available for 2 years, 2017 and 2018, so the 2-year average of the available years was used and the compliance rate was held constant for each year in the model since 2013.

The median value of a life-year was estimated to be $531,501 (2020 dollars), based on a comprehensive literature review of the value of a statistical life-year across 28 academic estimates, academic meta-analysis, and government agencies [30]. A sensitivity analysis was conducted using $150,000 per life-year.

## Results

### Population eligible for cancer screening

**Fig. 4:**
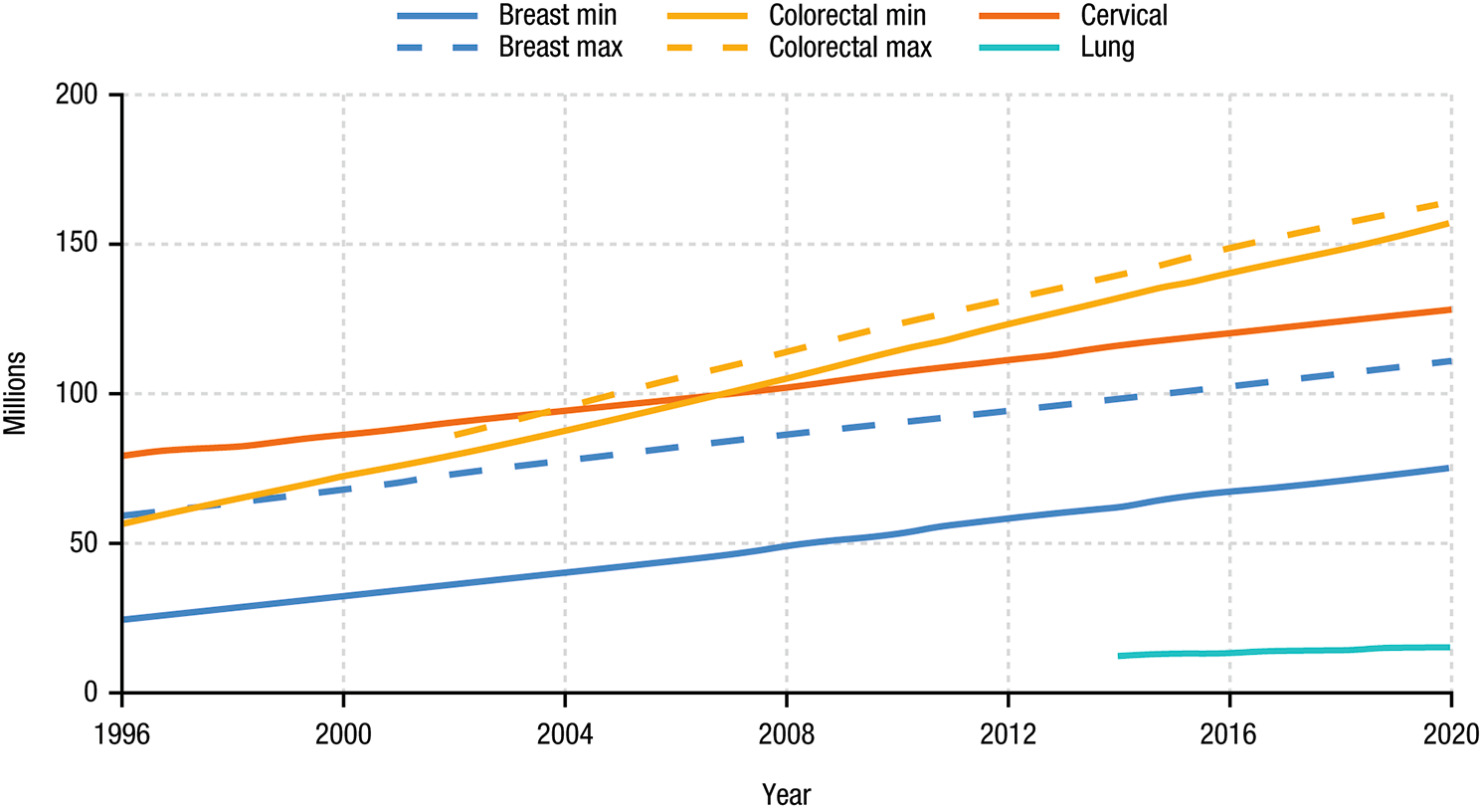
Cumulative Number of Eligible Individuals Since USPSTF Recommendations Max, maximum scenario; min, minimum scenario; USPSTF, US Preventive Services Task Force

The cumulative number of individuals eligible for screening in both minimum and maximum scenarios for each of the cancer types since USPSTF recommendations were made is shown in Fig. 4. In total, 375 to 417 million people were eligible for at least one type of cancer screening between 1996 and 2020. Colorectal cancer screening eligibility increased the most over time, adding about 4.2 million individuals annually, with a cumulative of > 150 million eligible individuals until 2020. The number of individuals eligible for lung cancer screening was the lowest among the 4 cancer types owing to the high-risk requirement from the USPSTF recommendation. The eligibility criteria for cervical and lung cancer were unchanged from the initial USPSTF recommendation until 2020; thus, the cohort sizes for the minimum and maximum scenarios are the same. Approximately 79.3 million males aged 50–74 have been eligible for PSA screening for prostate cancer since ACS recommendation in 1992.

### Adherence to cancer screening

**Fig. 5:**
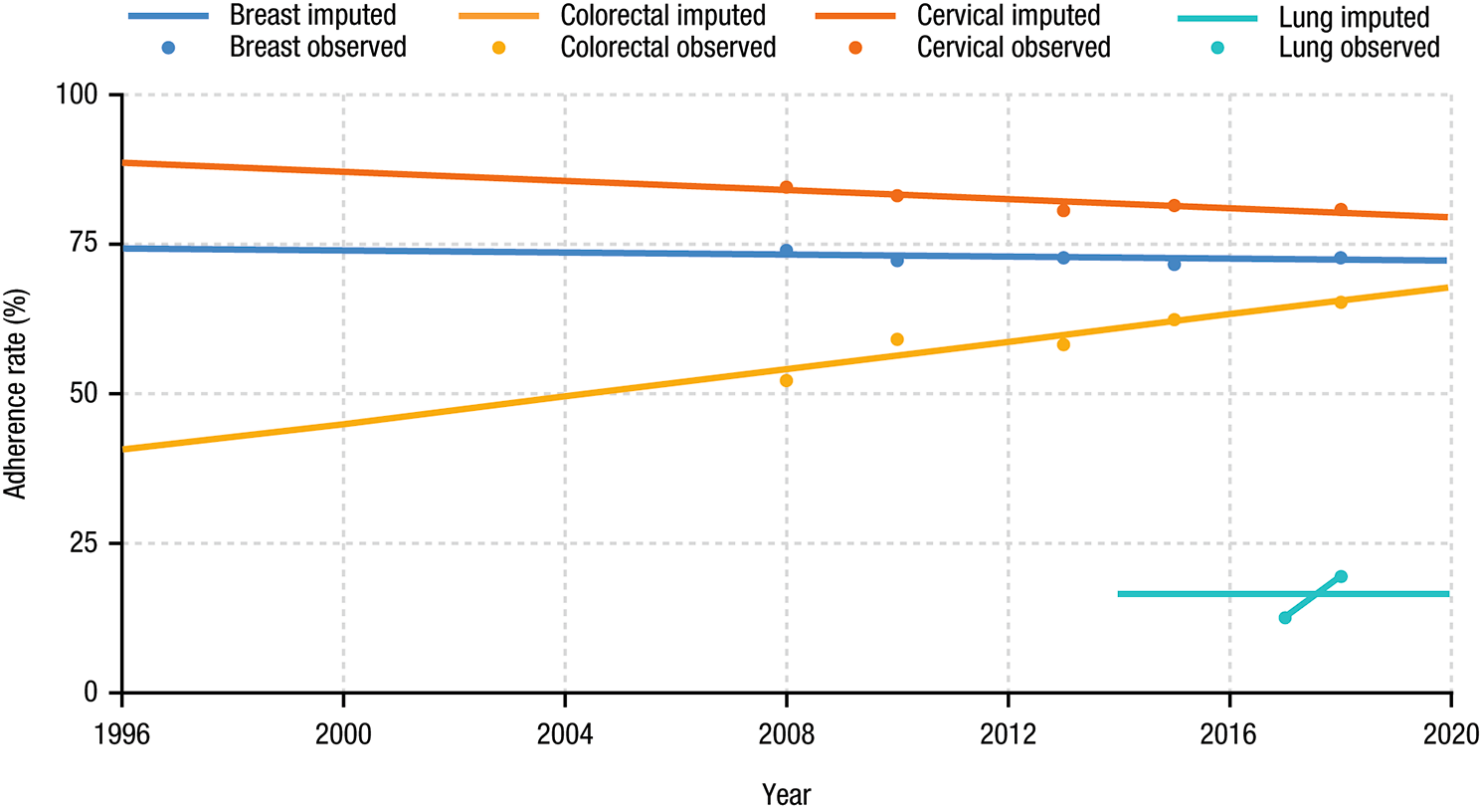
Observed and Imputed Cancer Screening Adherence Rates Over Time

Figure 5 shows adherence rates over time after imputation. Colorectal cancer has seen the highest increase in adherence rates, increasing from 52.1% to 2008 to 65.2% in 2018. Cervical cancer had the highest adherence rate over time but rates slightly decreased from 84.5% to 2008 to 80.5% in 2018, whereas breast cancer adherence rates have been fairly stable: 73.7% in 2008 and 72.8% in 2018. The observed adherence rate to PSA screening for prostate cancer has decreased from 64.6% to 1999 to 39.0% in 2008 due to the concerns of overdiagnosis and lack of evidence on the improvement in outcomes.

### Life-years gained

The total benefits of cancer screenings since USPSTF recommendations in terms of life-years gained and corresponding monetary value are shown in Table 2. With perfect adherence, the full potential number of life-years gained was estimated to be approximately 15.5 to 21.3 million. Of the 4 cancers, the greatest number of full potential life-years gained was for cervical cancer, which accounted for 58–74% of total benefits across all 4 cancer types (11.4 to 12.3 million life-years). The share attributed to breast cancer was 14–23% (2.2 to 4.9 million life-years), to colorectal cancer was 9–17% (1.4 to 3.6 million life-years), and to lung cancer was 2–3% (0.5 million life-years). The number of life-years gained is greatest for cervical cancer because this is one of the largest cohorts and cervical cancer screening has the highest effectiveness per screened individual according to the literature. Conversely, lung cancer has the smallest number of life-years gained due to small population size and low adherence to low-dose CT screening. When considering cancer screening adherence rates shown in Fig. 5, the total number of life-years gained was approximately 12.2 to 16.2 million across the 4 cancer types, or about three-quarters of the full potential benefit (Table 3). The full potential life-years gained from PSA for prostate cancer screening was estimated to be 4.4–5.1 million, whereas 2.4–2.8 million life-years could be saved considering PSA adherence.

**Table 2:** Full Potential Benefits and Associated Value Since USPSTF Recommendations

Cancer Type	Total Cohort Size of Eligible Individuals (millions of people)	Full Potential Life-Years Gained (millions)	Full Potential Value Life-Years Gained ($ trillions)
Breast	75–110	2.2–4.9	$1.2-$2.6
Colorectal	157–164	1.4–3.6	$0.8-$1.9
Cervical	128	11.4–12.3	$6.1-$6.5
Lung	15	0.5	$0.3
**Total**	**375–417**	**15.5–21.3**	**$8.2-$11.3**

USPSTF, US Preventive Services Task Force

**Table 3:** Benefits and Value of Cancer Screenings Since USPSTF Recommendations Considering Adherence

Cancer Type	Total Cohort Size of Eligible Individuals Considering Adherence (millions of people)	Life-Years Gained Considering Adherence (millions of life years)	Value of the Life-Years Gained Considering Adherence ($ trillions)
Breast	55–81	1.6–3.6	$0.8-$1.9
Colorectal	78–85	0.7–1.9	$0.4-$1.0
Cervical	111	9.9–10.6	$5.2-$5.7
Lung	2.4	0.08	$0.04
**Total**	**244–278**	**12.2–16.2**	**$6.5-$8.6**

USPSTF, US Preventive Services Task Force

### Value of life-years gained

Using the value of a statistical life-year of $531,501 based on a systematic review [30], we calculated the aggregate value of these screening technologies to be between $8.2 and $11.3 trillion at full potential (Table 2) and $6.5 to $8.6 trillion considering adherence rates (Table 3). According to these calculations, if all patients were perfectly adherent, these single-site screening technologies could have led to an additional 3.2 to 5.1 million life-years gained, equating to $1.7 to $2.7 trillion [30]. In a sensitivity analysis using a more conservative VLY of $150,000, the total value is estimated to be $2.3 to $3.2 trillion at full potential and $1.8 to $2.4 trillion considering adherence.

**Fig. 6:**
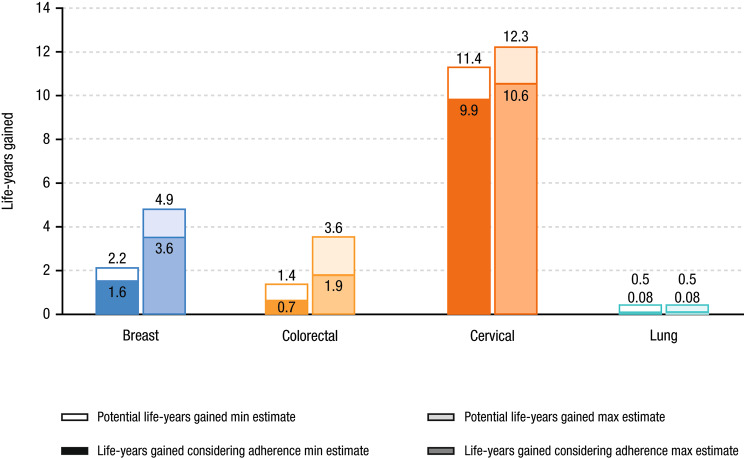
Full Potential Number of Life-Years Gained and Number of Life-Years Gained Considering Adherence Max, maximum; Min, minimum

**Fig. 7:**
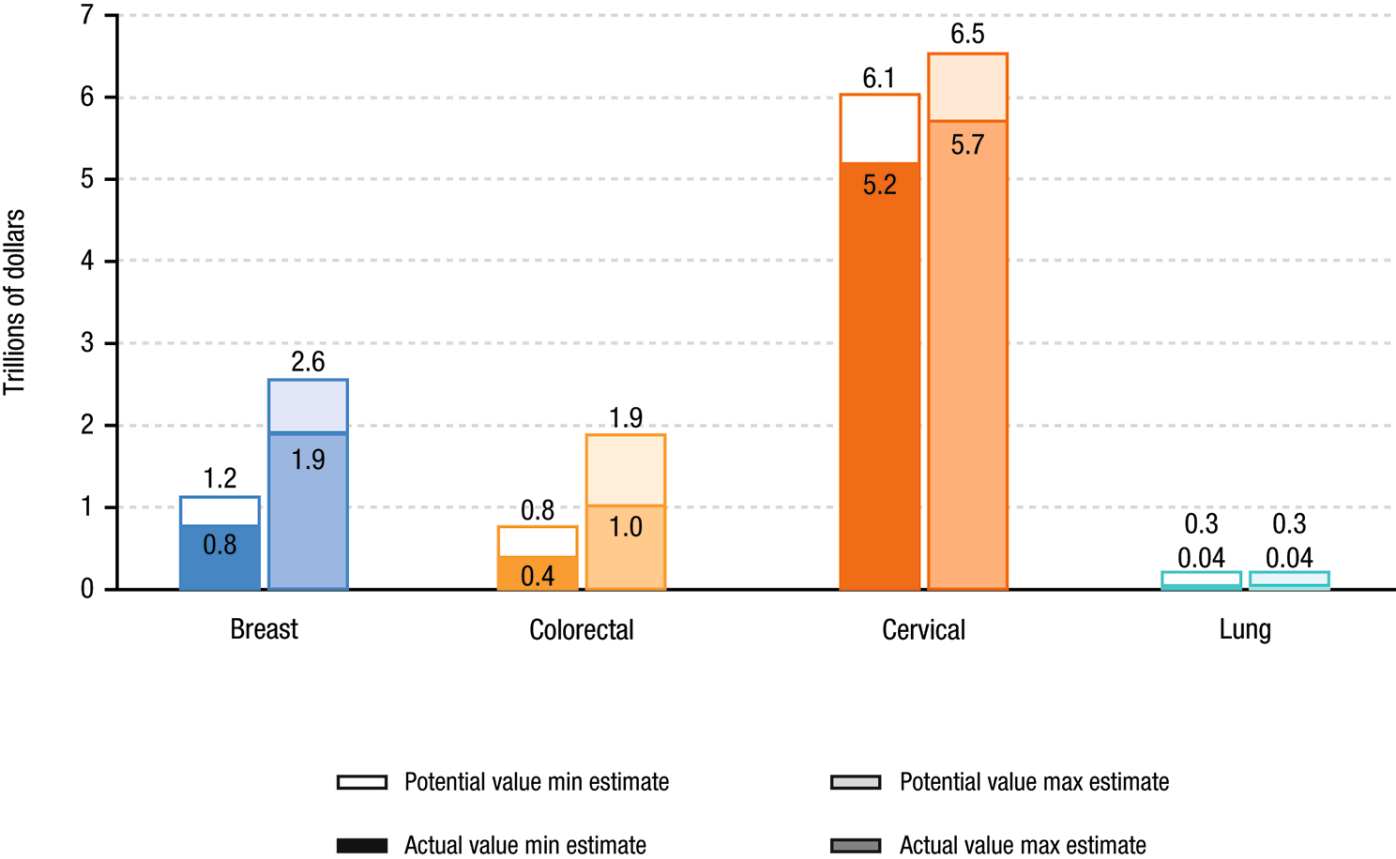
Full Potential and Value of Life-Years Gained Considering Adherence Max, maximum; Min, minimum

**Fig. 8:**
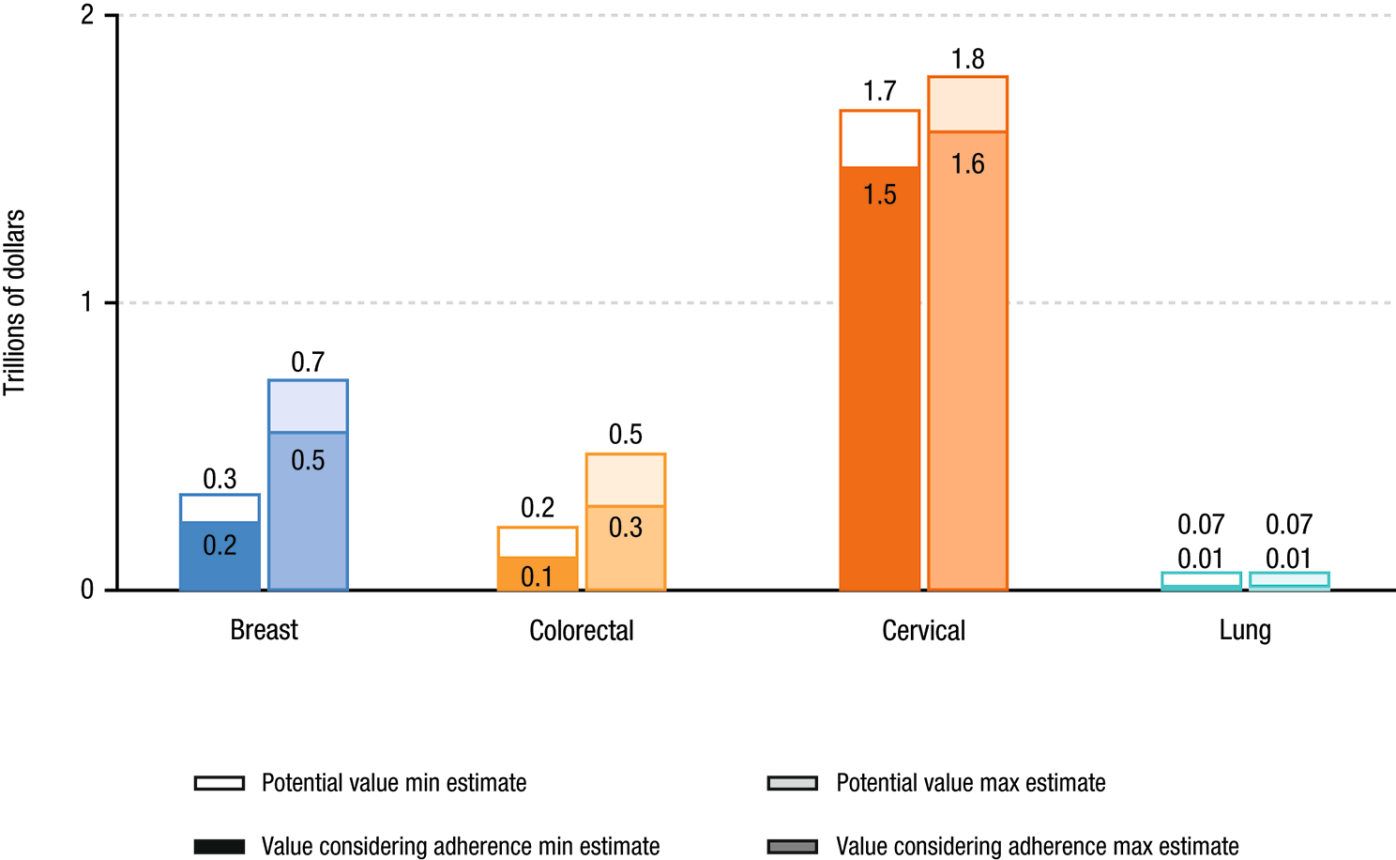
Full Potential and Value of Life-Years Gained Considering Adherence (Sensitivity Analysis)^a^ ^a^Sensitivity analysis using a VLY of $150,000 Max, maximum; Min, minimum; VLY, value of a life-year

Colorectal and breast cancer have the largest gaps between minimum and maximum estimates of potential life-years gained (2.2 and 2.7 million life-years, and 155% and 123%, respectively), in level and percent terms (Fig. 6). Lung cancer had the largest gap between potential life-years gained and life-years gained considering adherence (0.42 million life-years, 531%) due to the lowest adherence rate (Fig. 6). Cervical ($0.8 to $0.9 trillion) and colorectal ($0.4 to $0.9 trillion) cancer had the largest ranges between the full potential life-years gained and life-years gained considering adherence values in levels; cervical cancer had the smallest gap ($0.4 trillion; Fig. 7). The gap in prostate cancer was about $1 trillion. In the sensitivity analysis using a VLY of $150,000, breast ($0.3 to $0.4 trillion) and colorectal ($0.2 to $0.3 trillion) cancer had the largest ranges between the full potential life-years gained and life-years gained considering adherence values (Fig. 8).

## Discussion

The USPSTF recommendations for cancer screenings for breast, colorectal, cervical, and lung cancers have led to substantial positive outcomes for patients. That said, substantial additional gains (i.e., an additional 3.2 to 5.1 million life-years) are possible if adherence rates are optimized. Based on the results of the present study, the full potential benefits of screenings for these cancers have resulted in 15.5 to 21.3 million life-years gained, which translates into a value of $8.2 to $11.3 trillion since the start of the recommendation. With PSA for prostate cancer screening considered, the cumulative benefits could be even higher. This study builds upon previous evidence by providing an aggregate calculation of the total benefits for the 4 main types of cancer screening and examining the potential value if all eligible persons received screening [12, 36]. To our knowledge, this is the first study estimating the population-level, longitudinal, aggregate survival benefits of cancer screenings. This research helps to explain the observed trend in cancer mortality rate reduction in the Annual Report to the Nation on the Status of Cancer [37]. New multi-cancer early detection (MCED) blood tests that can simultaneously screen for multiple cancer types have been developed [38–40]. Given the recency of the technology, the value of MCED tests has not yet been fully quantified. This research will help provide benchmarks for the quantification of the potential value of MCED tests.

Due to less than perfect adherence, a non-trivial gap of 3.2 to 5.1 million life-years, equaling $1.7 to $2.7 trillion of value, exists between the full potential value and the value considering adherence rates, suggesting that one-quarter of the potential benefit goes unachieved. The lost value of the screenings highlights the importance of removing barriers to screening that prevent patients from being fully adherent with recommendations, including monetary screening cost, need for a specific clinic visit, procedure invasiveness, extra patient preparations prior to screening, or missed work days due to recovery after the procedure [17]. Patients may also be reluctant to undergo screening due to potential anxiety while awaiting test results [17, 41]. These and other barriers such as physicians’ recommendation on cancer screenings prevent individual screenings from reaching their full potential value.

The loss in life-years from coronavirus disease 2019 (COVID-19) was calculated by matching life expectancy estimates conditional on age from the Centers for Disease Control and Prevention to the age distribution of deaths from COVID-19 updated through August 2022 [42]. The average life expectancy at each age grouping was multiplied by deaths at each age grouping then summed, resulting in a finding that COVID-19 has directly reduced healthcare outcomes by 15.3 million life-years [30]. The life-years gained considering adherence from early screening for breast, colorectal, cervical, and lung cancer is 12.2 to 16.2 million years, or about 0.8 to 1.1 times the life-years lost to COVID-19, whereas the full potential life-years gained is 1.0 to 1.4 times larger.

Cervical cancer screening is estimated to offer the largest estimated benefit in life-years gained due to the USPSTF recommendation that women start cervical cancer screening at a young age (i.e., aged 21 years, more than 2 decades before recommended screening for any other cancer), the high effectiveness of cervical cancer screening [43], and the high adherence rates. Indeed, although cervical cancer was previously a leading cause of death for women in the United States [44], mortality from cervical cancer dropped by 60% in recent decades, from a death rate of 5.5 per 100,000 persons in 1975 to 2.2 per 100,000 persons in 2022 [45], with probably even higher rates before widespread screening with the Papanicolaou smear began in the 1940s [43].

Modeling data have estimated that MCED tests could provide approximately 0.18 life-year gained per tested individual by screening for more than 50 types of cancer, in addition to available cancer screening tests [46]. The 5-year full potential benefits of MCED tests assuming perfect adherence were estimated to be more than 23 million life-years gained, which is greater than the total benefits across the screenings for the 4 cancer types assessed in this study. The difference in the value considering adherence and the full potential value of early cancer screenings shown in this study suggests that additional value could be provided by new technologies, such as MCED [39, 40], or policy interventions directed to remove barriers and improve adherence to existing cancer screenings or expand the number of cancer types screened, because many aggressive cancers do not yet have a screening protocol recommended by the USPSTF.

This study has limitations. First, when calculating the value of screening by considering adherence, both upward and downward bias exists because adherence is assumed constant for a patient’s full time in the recommended screening age. Upward bias exists because many patients start out fully adherent, but over time, they stop following the USPSTF’s screening recommendations. In this case, our methodology overestimates the value considering adherence. However, downward bias exists because many patients may start out nonadherent but, over time, they begin to follow the USPSTF’s screening recommendations. In this scenario, our methodology underestimates the value gained considering adherence.

Second, each scenario assumes a constant life-years-gained estimate for each of the screenings, but it is more likely that the size of this estimate changes over time with drug and technology innovation, which can influence relative mortality through earlier cancer detection, and subsequently alter the effectiveness of screening due to advances in either early- or late-stage cancer treatments. For example, human papillomavirus (HPV) infections are a clear predictor of cervical cancer, so introduction of the HPV vaccine in 2006 may alter the effectiveness of cervical cancer screening as more people receive the vaccine and do not become infected with HPV [13]. Furthermore, each patient may have different life-years gained depending on age and whether they are at the youngest or oldest end of the recommended age group for screening.

Recommendations for different screenings have changed over time, which influences the number of individuals eligible for screening. Due to changing recommendations, our methodology applies the most restrictive population (smallest cohort) to the smallest life-years gained and least restrictive population (largest cohort) with the largest life-years gained, to provide a range of scenarios that account for such changes. The subsequent range of outcomes reflects the extremes of a small population-low life-years gained and a large population-high life-years gained, due to different screening recommendations. A tradeoff exists between the number of eligible patients and the benefits per screened individual. In other words, the total value of screening may increase by increasing the number of individuals screened, but the effectiveness of each individual test will likely decrease.

Third, the model inputs of this research analysis are mostly based on literature, hence the bias and limitations from the referenced studies contribute to the basis of our research. For example, in terms of life-years gained per screened individual, the referenced studies are all cost-effectiveness modeling research with underlying assumptions. By using estimates from those studies, this research is inherently making those assumptions as well.

Finally, adherence rates used in this analysis only accounted for initial screening. For example, a patient receiving a positive result from an initial screening often undergoes additional tests, such as biopsy, to confirm an official cancer diagnosis. Costs and other barriers likely lead the resulting adherence to the full diagnostic procedure to be lower than the NHIS estimates [47–49]. Patients may not receive the full benefit of cancer screening if they get a diagnosis of cancer (or not), making the total number of screened individuals an upper bound estimate. Of note, the types and frequency of recommended cancer screening tests differ for individuals based on health status and physician recommendation.

## Conclusions

Single-site cancer screenings have offered significant cumulative gains to US life-years gained and improvements to value of screening, despite screening adherence leading to a nontrivial gap between full potential and realized benefit considering adherence. These analyses suggest that technologies and policy interventions that can improve adherence to existing screening and/or expand the number of cancer types screened for, will provide significant value.

### Electronic supplementary material

Below is the link to the electronic supplementary material.

Supplementary Material 1

Supplementary Material 2

## Data Availability

The datasets used and/or analyzed during the current study are available from the corresponding author on reasonable request. All data generated or analyzed during this study are included in this published article (direct references to source data are included in Supplemental Table 2).

## Declarations

### Author information

TJP is the Daniel Levin Professor of Public Policy Studies Emeritus at the University of Chicago Harris School of Public Policy. AMF is a professor at the division of General Medicine, Department of Internal Medicine and Department of Health Management and Policy at the University of Michigan. He is also the director of the University of Michigan Center for Value-Based Insurance Design.

## Abbreviations

BRFSS: Behavioral Risk Factor Surveillance System
COVID-19: Coronavirus disease 2019
CT: Computed tomography
HPV: Human papillomavirus
MCED: Multi-cancer early detection
NHIS: National Health Interview Survey
USPSTF: US Preventive Services Task Force
VLY: Value of a life-year
