# Supplementary material for: The aggregate value of cancer screenings in the United States: full potential value and value considering adherence
Source: BMC Health Serv Res. 2023 Aug 7;23:829. doi: 10.1186/s12913-023-09738-4 (PMC10405449; doi:10.1186/s12913-023-09738-4)
Supplement: Supplementary file 1 — Supplementary Material 1 [file 12913_2023_9738_MOESM1_ESM.docx]

**Supplemental Table 1.**

**Assumptions in Life-years Gained From Cancer Screening Literature**

| **Cancer type** | **Breast** | | **Colorectal** | | **Cervical** | | **Lung** | **Prostate** | |
| --- | --- | --- | --- | --- | --- | --- | --- | --- | --- |
| **Scenario** | Minimum | Maximum | Minimum | Maximum | Minimum | Maximum | Minimum and Maximum | Minimum | Maximum |
| **Reference** | Shih et al 2021 [1] | | Barzi 2017 [2] | | Goldie 2004 [3] | | Black 2014 [4] | Roth 2016 [5] | |
| **Effectiveness (LY gained per screened individual)** | 0.029 | 0.044 | 0.009 | 0.022 | 0.089 | 0.096 | 0.032 | 0.055 | 0.064 |
| **Model type** | Microsimulation | | Markov model | | State transition mathematical model | | Trial based analyses | Microsimulation | |
| **Time horizon** | Lifetime | | Lifetime | | Lifetime | | 3 years | Lifetime | |
| **Annual Discount** | 3% | | 3% | | 3% | | 3% | 3% | |
| **Screening intervention** | Mammography, biennial | Mammography, annual for dense breast, biennial for others | Flex sig, every 5 years and FIT, annual | Colonoscopy, every 10 years (every 3 or 5 years with adenoma) | PAP, every 3 years | PAP, every 3 years until age 30, combination of PAP and HPV after age 30 | LDCT, annually for 3 years | PSA, annual for age 50-74, PSA threshold for biopsy=10 μg/L | PSA, annual for age 50-74, PSA threshold for biopsy=4 μg/L |

LY, life-years; Flex sig, flexible sigmoidoscopy; FIT, fecal immunochemical test; PAP, pap smear; HPV, human papillomavirus; LDCT, low-dose computed tomography; PSA, prostate specific antigen.

**References**

1. Shih YT, Dong W, Xu Y, Etzioni R, Shen Y. Incorporating Baseline Breast Density When Screening Women at Average Risk for Breast Cancer : A Cost-Effectiveness Analysis. Ann Intern Med. 2021;174:602-12.

2. Barzi A, Lenz HJ, Quinn DI, Sadeghi S. Comparative effectiveness of screening strategies for colorectal cancer. Cancer. 2017;123:1516-27.

3. Goldie SJ, Kim JJ, Wright TC. Cost-effectiveness of human papillomavirus DNA testing for cervical cancer screening in women aged 30 years or more. Obstet Gynecol. 2004;103:619-31.

4. Black WC, Gareen IF, Soneji SS, Sicks JD, Keeler EB, Aberle DR, et al. Cost-effectiveness of CT screening in the National Lung Screening Trial. N Engl J Med. 2014;371:1793-802.

5. Roth JA, Gulati R, Gore JL, Cooperberg MR, Etzioni R. Economic Analysis of Prostate-Specific Antigen Screening and Selective Treatment Strategies. JAMA Oncol. 2016;2:890-8.
