# Supplementary material for: The aggregate value of cancer screenings in the United States: full potential value and value considering adherence
Source: BMC Health Serv Res. 2023 Aug 7;23:829. doi: 10.1186/s12913-023-09738-4 (PMC10405449; doi:10.1186/s12913-023-09738-4)
Supplement: Supplementary file 2 — Supplementary Material 2 [file 12913_2023_9738_MOESM2_ESM.docx]

**Supplemental Table 2**. Source Data

| **Model Inputs** | **Source** |
| --- | --- |
| Size of eligible population | US Census Bureau’s Vintage annual national population estimates by demographic characteristics and US Census National Intercensal Datasets (<https://www.census.gov/en.html>) |
| Proportion of adults aged 55 to 80 who are eligible for lung cancer screening | BRFSS survey in 2017 [1] |
| Time period and eligibility for cancer screening | USPSTF recommendations [2-8] |
| Effectiveness of cancer screenings in terms of life year gained per screened individual | Literature [9-13] |
| Adherence rate to USPSTF recommendations for cancer screening | National Health Interview Survey in 2008, 2020, 2023, 2015, and 2018 with extrapolation for breast, cervical, and colorectal cancer screenings [14]  BRFSS survey in 2017 and 2018 for lung cancer screening [1, 15] |

**References**

1. Richards TB, Soman A, Thomas CC, VanFrank B, Henley SJ, Gallaway MS, Richardson LC: Screening for Lung Cancer - 10 States, 2017. *MMWR Morb Mortal Wkly Rep* 2020, 69(8):201-206.

2. US Preventive Services Task Force, Davidson KW, Barry MJ, Mangione CM, Cabana M, Caughey AB, Davis EM, Donahue KE, Doubeni CA, Krist AH *et al*: Screening for colorectal cancer: US Preventive Services Task Force recommendation statement. *JAMA* 2021, 325(19):1965-1977.

3. U.S. Preventive Services Task Force, Curry SJ, Krist AH, Owens DK, Barry MJ, Caughey AB, Davidson KW, Doubeni CA, Epling JW, Jr., Kemper AR *et al*: Screening for cervical cancer: US preventive services task force recommendation statement. *JAMA* 2018, 320(7):674-686.

4. US Preventive Services Task Force, Krist AH, Davidson KW, Mangione CM, Barry MJ, Cabana M, Caughey AB, Davis EM, Donahue KE, Doubeni CA *et al*: Screening for lung cancer: US preventive services task force recommendation statement. *JAMA* 2021, 325(10):962-970.

5. Breast Cancer: Screening [<https://www.uspreventiveservicestaskforce.org/uspstf/recommendation/breast-cancer-screening>]

6. Breast cancer: screening, 1996 [<https://www.uspreventiveservicestaskforce.org/uspstf/recommendation/breast-cancer-screening-1996>]

7. Breast cancer: screening, 2002 [<https://www.uspreventiveservicestaskforce.org/uspstf/recommendation/breast-cancer-screening-2002>]

8. Breast cancer: screening, 2009 [<https://uspreventiveservicestaskforce.org/uspstf/recommendation/breast-cancer-screening-2009>]

9. Tina Shih YC, Dong W, Xu Y, Shen Y: Assessing the cost-effectiveness of updated breast cancer screening guidelines for average-risk women. *Value Health* 2019, 22(2):185-193.

10. Barzi A, Lenz HJ, Quinn DI, Sadeghi S: Comparative effectiveness of screening strategies for colorectal cancer. *Cancer* 2017, 123(9):1516-1527.

11. Goldie SJ, Kim JJ, Wright TC: Cost-effectiveness of human papillomavirus DNA testing for cervical cancer screening in women aged 30 years or more. *Obstet Gynecol* 2004, 103(4):619-631.

12. Black WC, Gareen IF, Soneji SS, Sicks JD, Keeler EB, Aberle DR, Naeim A, Church TR, Silvestri GA, Gorelick J *et al*: Cost-effectiveness of CT screening in the National Lung Screening Trial. *N Engl J Med* 2014, 371(19):1793-1802.

13. Philipson T, Durie T: Issue brief: The evidence base on the impact of price controls on medical innovation. In*.*; 2021.

14. National health interview survey [<https://www.cdc.gov/nchs/nhis/index.htm>]

15. Narayan AK, Gupta Y, Little BP, Shepard JO, Flores EJ: Lung cancer screening eligibility and use with low-dose computed tomography: Results from the 2018 Behavioral Risk Factor Surveillance System cross-sectional survey. *Cancer* 2021, 127(5):748-756.
